# Military Medicine and Medical Research as a Source of Inspiration and Innovation to Solve National Security and Health Challenges in the 21st Century

**DOI:** 10.20411/pai.v8i1.596

**Published:** 2023-09-08

**Authors:** Nanak S. Dhillon, Nayeon Jeon, Umut A. Gurkan, Anirban Sen Gupta, Robert A. Bonomo, Lawrence F. Drummy, Mei Zhang, Mark R. Chance

**Affiliations:** 1 Department of Nutrition, School of Medicine, Case Western Reserve University, Cleveland, OH; 2 Center for Proteomics and Bioinformatics, School of Medicine, Case Western Reserve University, Cleveland, Ohio; 3 Department of Mechanical and Aerospace Engineering, Case School of Engineering, Case Western Reserve University, Cleveland, Ohio; 4 Department of Biomedical Engineering, School of Medicine, Case School of Engineering, Case Western Reserve University, Cleveland, Ohio; 5 Louis Stokes Cleveland Department of Veterans Affairs Medical Center; Case Western Reserve University, Cleveland, OH; VAMC Center for Antimicrobial Resistance and Epidemiology (Case VA CARES); Departments of Medicine, Pharmacology, Molecular Biology and Microbiology, and Biochemistry, Case Western Reserve University, Cleveland, Ohio; 6 Materials and Manufacturing Directorate, Air Force Research Laboratory, Dayton, Ohio

**Keywords:** Health research, fatigue and stress, sensors, human performance and resilience

## Abstract

The history of military medicine and research is rife with examples of novel treatments and new approaches to heal and cure soldiers and others impacted by war's devastation. In the 21st century, new threats, like climate change, are combined with traditional threats, like geopolitical conflict, to create novel challenges for our strategic interests. Extreme and inaccessible environments provide heightened risks for warfighter exposure to dangerous bacteria, viruses, and fungi, as well as exposure to toxic substances and extremes of temperature, pressure, or both providing threats to performance and eroding resilience. Back home, caring for our veterans is also a health-care priority, and the diseases of veterans increasingly overlap with the health needs of an aging society. These trends of climate change, politics, and demographics suggest performance evaluation and resilience planning and response are critical to assuring both warfighter performance and societal health. The Cleveland ecosystem, comprising several hospitals, a leading University, and one of the nation's larger Veteran's Health Administration systems, is ideal for incubating and understanding the response to these challenges. In this review, we explore the interconnections of collaborations between Defense agencies, particularly Air Force and Army and academic medical center-based investigators to drive responses to the national health security challenges facing the United States and the world.

## INTRODUCTION

Department of Defense (DOD)-related biomedical research is a large and growing enterprise, and with the recent formation of the Advanced Research Projects Agency for Health, it is expanding its potential for additional funding of relevant projects [1, 2]. Academic medical centers are receiving tens of billions of dollars in federal funding to find cures and treatments for diseases, and programs are being developed to help investigators learn to transfer these innovations from the laboratory to the hands of patients and providers [3, 4]. In particular, the US National Biodefense Strategy (Oct 2022) recognizes that pathogens are global risks, and that enhancing resilience means strengthening global health defense to protect the nation in the same ways we develop and project conventional defenses. We cannot start to fight at the border because that will be too late. A DOD-inspired symposium was recently held at Case Western Reserve University in Cleveland, Ohio, where field-leading experts from all over the country were able to convene and discuss some of the challenges and innovations outlined in this paper.

In examining the potential future needs for protecting the United States and its warfighters and their support systems, a few obvious trends are settling in place. First, the trend of climate change will increase the frequency of serious infections, extend the ranges of some pathogen-induced diseases, and escalate the severity of heat-related threats to warfighters and society. Second, current geopolitical conflict trends suggest that many battles are fought in unfamiliar, extreme, or challenging environments where monitoring performance in real time and enhancing resilience may be critical to success. Lastly, an aging US population and an aging veteran population increasingly merge the needs of veterans and society at large. Integrating disparate federally supported research efforts can only help to improve efficiencies and increase opportunities for collaborations to permit faster and further progress toward health solutions. In this review, the authors outline their individual and joint efforts to respond to these emerging challenges. In total, these activities intend to measure and optimize human performance, and develop warfighter and societal resilience, both in advance of threats and in response to them.

## HISTORY OF MEDICINE IN WARFARE

To understand the future of military medicine and the value of biomedical research to the welfare of soldiers and veterans, we must first understand its past. Critical to understand is the concept of *War Pestilence*, in which infectious diseases claimed the lives of many troops. In fact, diseases such as malaria, typhoid, plague, dysentery, and cholera caused approximately 7 times as many deaths as combat-related injuries in the period prior to the 19th century [5, 6]. In response to this obvious need, there is an extensive history of wound and infection care dating back to over 4,000 years ago. In this period, according to stone carvings, Sumerians used beer and milk to wash wounds before wrapping them [7]. Approximately 3,500 years ago, the Egyptians used the antibacterial properties of honey to treat wounds. Approximately 2,800 years ago, the Greeks used hot water and wine to clean wounds [8]. As gunpowder became commonly used in 14th and 15th century Europe, wounds became much more complex to treat. In the American Civil War, the recent development of general anesthesia played a crucial role in delaying amputation to reduce the effect of wound shock, and bromine was used to prevent hospital gangrene [9]. An estimated 60,000 amputations were performed in the newly developed pavilion-type hospitals [10].

In World War I, the critical developments of debridement, ABO-compatible blood transfusions, and topical antiseptics saved many lives [11]. Additionally, an important observation on the types of bacteria found in wounds in World War I, is that these pathogens were primarily Gram-positive, and relatively simple to understand. In World War II, sulfanilamide and penicillin were used to treat the majority of bacterial infections. In the Korean War, the innovation of Mobile Army Surgical Hospitals (MASH) and the use of helicopters for rapid evacuation of casualties helped save many lives [12]. Towards the end of the Korean war, penicillins and streptomycin were used for bacterial infections, and were particularly effective within the “golden hour,” or first 60 minutes, but some resistance began to be detected.

By the Vietnam War, there was a rapid evolution seen in the complexity of bacterial pathogens, as a mixture of both Gram-positive and Gram-negative bacteria became prevalent [13]. This rapid evolution foreshadowed what would then happen in Iraq and Afghanistan, with even more complex bacteria becoming predominant (ie, *Acinetobacter* spp.) Entering the modern day, there are many multi-drug resistant (MDR) bacterial strains that are emerging [14]. The Military Infectious Diseases Research Program (MIDRP) accordingly has a goal of helping the DOD prevent, predict, and treat these evolving disease threats. Vector-borne and zoonotic threats have become increasingly threatening to troops [15]. In modern warfare, explosive injuries and resulting infections are of key concern.

All the above provide concrete examples of military-driven innovation that became standard practice for use in society shortly thereafter. Going forward, the focus of infectious disease programs (eg, MIDRP) includes wound infections, endemic diarrheal diseases, viral diseases including HIV, dengue virus, and emerging infectious diseases [16]. By predicting, preventing, and treating these threats, more warfighters can stay on the battlefield, and more support personnel can continue maintaining combat power and increasing battle success rates.

**Table 1.** Three Thousand Year Timeline of War Periods and Corresponding Medical Innovations

**Common source of infectious diseases**	Spear wounds	Gunshot wounds	Wound shock	Gram positive bacteria	Gram positive bacteria	Bacterial resistance detected	Mix of gram positive, negative bacteria	Multidrug resistant (MDR) bacteria
**War/Time Period**	Civilizations in Centuries BC	Europe 14th Century	Civil War	WWI	WWII	Korean War	Vietnam War	Modern Day
**Innovation**	Alcohol, hot water, honey	Amputation	General anesthesia, bromine. Pavilion hospitals.	Debridement, ABO blood transfusion, topical antiseptics, x-rays	Sulfanilamide, penicillin, masks and sterile instruments	Mobile Army Surgical Hospitals (MASH), streptomycin	Rapid helicopter evacuation and better surgeon training	Military Infectious Disease Research Program (MIDRP)

## CLIMATE CHANGE AND COVID-19: INFECTIOUS DISEASE RISK AND RESEARCH

A hypothesis developed from the work of Arturo Casadevall may explain why we are seeing a rapid evolution in disease threats over a relatively short period of time [17]. The hypothesis is that climate change is playing a pivotal role in accelerating the evolution of infectious diseases by increasing the frequency of cross-species transmission [18]. As many environments are becoming less suitable for animal and human life due to natural disasters, deforestation, and temperature shifts, the environments of animals and humans are being pushed closer together. As animal-animal and human-animal interactions become more frequent, vector-borne diseases are having more opportunities to travel between species. This increased transmission is allowing for re-emergence of previously diminished infection threats, the evolution of infection threats, and the emergence of entirely new threats like COVID-19 [19]. Thus, the need to increase prioritization of vaccines and other means of subverting viral, bacterial, fungal, and parasitic pathogens is apparent. Collaboration of global health and epidemiology departments coupled with immunology and pharmacology research programs are prime candidates for inclusion on relevant research teams. Host defense and immunity will play increasing roles in disease management and treatment. The development of warfighter agents will also overlap with civilian needs, emphasizing the logic of translational initiatives that benefit both the DOD and society at large.

## BIOMARKERS FOR HUMAN PERFORMANCE

Performance monitoring of warfighters raises the question: What measurements are actionable for real-world warfighter situations? The question depends on the application of course, but models for examining performance can be derived from high stress and fatigue situations such as combat aviation. Damato and colleagues have suggested that “cognitive fatigue” is an outcome of tactical aviation-induced systemic inflammation [20]. They hypothesized the relationships between serum analytes and “brain fog” in aviation, which could be potential biomarkers of fatigue and sleepiness for tactical aviators. For this study, brain fog was defined as both fatigue and sleepiness, which was repeatedly reported by instructor pilots who fly multiple flights per day with student pilots. This is a constant threat for aviators, particularly because it is the “likely cause of the next mishap,” endangering aviator safety and reducing human performance. Researchers must identify the quantitative physiological biomarkers that correspond to increased levels of fatigue to prevent accidents and enhance flight performance. For tactical aviators, both fatigue and sleepiness appear upon increased levels of proinflammatory cytokines in systemic and central nervous systems. Due to frequent exposure to high-performance aviation events, recurring synthesis and release of proinflammatory cytokines may induce cognitive fatigue in Air Force pilots.

To confirm potential relationships between the biochemical mechanisms and the rise of fatigue for pilots, T-6A Texan II instructor pilots who were scheduled to have more than 2 flights during data collection week were examined. Damato et al made a physical assessment and general, physical, and mental fatigue, motivation and activity, as well as urine, serum, and blood chemistry analysis. Eleven serum analytes were significantly correlated with increased levels of general fatigue, including MCP-1 and MCP-4. These monocyte chemoattractant proteins play a key role in recruiting inflammatory cells to inflammation sites, by activating cell migration signaling pathways [21]. Beyond general fatigue, they have also been implicated in neuroinflammatory and cardiovascular diseases. The data from Damato et al provides both potential biomarkers, as well as mechanistic information on fatigue and stress. However, there is a gap in correlating these novel molecular markers with traditional performance measures that can only be filled by relevant clinical studies. Over time, these biomarker and clinical data will be merged with traditional measures of fitness and readiness and further coupled to additional physiologic data (like blood pressure, heart rate, etc.) to provide multi-modal performance “instruments” to help assist in flight readiness decision making. Further, the need for performance sensing is also significant in the civilian community, as sensors for fatigue could have significant utility for long-haul truck drivers, medical professionals, athletes, airline pilots, and others who experience high occupational stress, fatigue, or both. Real-time performance data can be applied to prevent fatigue-related traffic accidents, improve work efficiency, reduce occurrence of workplace injury, monitor injury recovery, and potentially much more. Performance monitoring is clearly a health challenge for not only the military, but also society at large, and with continued development of these devices that sense performance biomarkers such as lactate, there are opportunities to save lives and improve societal health [22].

## SENSORS FOR WARFIGHTER PERFORMANCE MONITORING

A primary focus of laboratory research for military medicine is to develop sensors for monitoring the health, safety, and performance of military personnel. The current status of diagnosis and monitoring using micro-technologies with point-of-care performance has recently been reviewed for COVID-19, but it has been noted that these devices may have many more profound disease applications as well [23]. An and colleagues have used anemia detection and hemoglobin variant identification as examples, as these are both key indicators of malaria and hemoglobinopathies such as sickle cell disease [24]. Particularly in low-resource settings where these diseases are also quite prevalent, the main challenges are cost, laboratory infrastructure, trained personnel, result interpretation, and data management [25]. Even in settings with ample resources, many of these challenges persist to a considerable extent. Nevertheless, a trend is emerging where physiologic types of signals (heart rate, blood pressure etc.) generated from wearables like rings, bands, or watches can now be supplemented with direct molecular measures of substances of interest in body fluids, for example, lactate, cortisol, or neuropeptide-Y, which reflect multiple potential health and disease states [26]. Thus, the limited prediction variables currently provided by physiologic monitoring will be revolutionized by the addition of specific molecular markers for understanding pathologies of interest.

Radwan et al have published on developments in electrochemical molecular biosensors and highlighted the importance of these sensors being highly specific to differentiate compounds with similar structures, as well as highly sensitive to accurately quantify the low levels found in biological analytes [27]. Additionally, these sensors must be simple to use and able to be miniaturized, give readings in real time, and communicate with other devices in a smart system or body-area network. Microneedles properly layered and prepared are for detecting substances such as cortisol, whose levels in serum parallel those seen in interstitial fluid [28]. Nanostructures deposited onto the surface of microneedles allow for the attachment of specific recognition elements that are capable of accurately sensing stress marker levels. These technologies have obvious applications for both warfighters and society, ranging from diagnostics in low-resource settings to real-time sensing of individuals, from athletes to patients in clinical trials.

**Figure 1.**
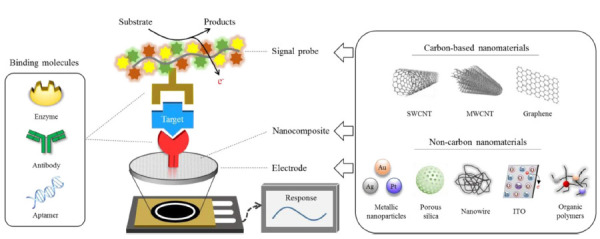
**Components of an electrochemical biosensor.** Electrochemical biosensors must be specific to their target to accurately characterize amidst signal noise as well as highly sensitive to precisely quantify the µM level concentrations found in biological samples. The necessary specificity and sensitivity are achieved by careful selection of surface molecular receptors and adjustment of enzymatic activity. Carbon-based electrodes offer high surface area to volume ratio, as well as rapid electron transfer capability, making them suitable for achieving miniaturization and real-time data output. [29]. *Image Source:* Cho IH, Kim DH, Park S. In *Biomaterials Research*. Springer Nature. Feb 4, 2020. CC BY 4.0.

## MODERN APPROACHES TO TRAUMA: ENHANCING WARFIGHTER RESILIENCE

Warfighter injuries like trauma and bleeding are immediate risks of combat, and the availability of blood and blood products is critical to saving injured personnel. The management of uncontrolled hemorrhage is crucial at warfare scenes. Acidosis, trauma-induced coagulopathy (TIC), and hypothermia, called the “Triangle of Death,” feed into each other to induce the death of soldiers [30]. These are worthy of attention, especially because they are the major causes of preventable mortality. As robust clinical research indicates the importance of early blood transfusion to reduce mortality, battlefield blood transfusion plays a vital role in the prevention trauma-induced hemorrhage and coagulopathy [31]. For transfusion on the battlefield, Anirban Sen Gupta and colleagues are currently adapting the Massive Transfusion Protocol that utilizes a 1:1:1 ratio mixture of platelet, red blood cells, and plasma. However, blood transfusion at military treatment facilities or pre-military treatment facilities faces challenges because of the difficulty in availability, portability, storage, and shelf-life of platelets [32]. Platelets are highly limited in availability in battlefield settings and require special requirements of temperature, container, and additive solution. They are also at higher risk of bacterial contamination and can only stay for 5 to 7 days at room temperature. To improve the availability of platelets, Sen Gupta proposed SynthoPlate, a synthetic platelet, which could significantly reduce bleeding time and quickly stabilize blood pressure by enhancing the aggregation of activated platelets [33]. Dual-use (or application to both civilian and military uses) of this product in society at large may have a significant impact. This is because rural areas, more than an hour from a major medical center where blood supplies for transfusion are most available, probably cannot treat a major trauma case, and helicopter evacuation may be the only option.

**Figure 2.**
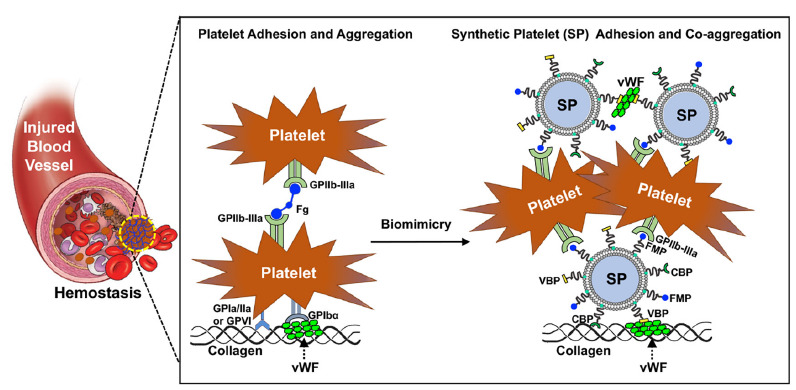
**SynthoPlate mechanism of hemostatic activity.** Platelets are cells that facilitate stoppage of bleeding (hemostasis) from injured blood vessels by forming a plug at the injury site via binding to specific proteins like Von Willebrand Factor (VWF) and collagen exposed at the site as well as by aggregating among each other via cross-linking of their surface integrin GPIIb-IIIa by fibrinogen (Fg). Synthetic Platelet (SP) systems are nanoparticles that mimic these platelet mechanisms and thus facilitate hemostasis by virtue of VWF-binding peptides (VBP), collagen-binding peptides (CBP), and Fg-mimetic peptides (FMP) combinatorially decorated on the particle surface.

## CANCER RESEARCH FOR THE DOD MISSION

Beyond specific mission readiness research pertinent to the needs of veterans and society at large, the DOD funds cancer research directed by Congressional mandates [34]. Since 1992, with the establishment of the Breast Cancer Research Program (BCRP), the DOD has allocated billions of dollars to cancer research programs, with a goal of improving the lives of service members and the civilian population [35]. These programs are focused on the most innovative types of treatment including a tagline of “Transforming Healthcare Through Innovation and Impactful Research.” In the cancer domain, invariably, this means treatments that lead to cures. Activating the host's own immune system to cure cancer is one of the most promising approaches of the last decade [36].

A recent development has been cancer-targeting cells engineered from the patient's own immune system, the T-cells, which, when re-injected in patients in a single dose, can cure many types of blood cancers [37]. These specially engineered T-cells, called chimeric antigen receptor T-cells or CAR-T, have shown the ability to cure patients by targeting a special signal called an epitope residing in the target cancer cells present in blood diseases like B-cell lymphoma or multiple myeloma [38]. The engineered CAR-T recognizes the cancer cell and its CD19 epitope signal and signals the host's immune system to fight and, in many cases, even kill the cancer, providing long-lasting and durable protections for patients. However, if the cancer cell loses its epitope signal, it can evade the CAR-T, with recurrence of cancer [39]. A novel BAFF-CAR-T, which targets 3 sites unique to cancer cells, including a target called BAFF, which is under development in collaboration with Luminary Therapeutics to treat multiple myeloma and lymphoma patients [40].

Another cancer immune therapeutic approach that has also shown great promise is to target immune checkpoint proteins such as PD-1, PD-L1, and CTLA-4 [41]. These checkpoint proteins enable malignant cells to pass through the body undetected, by disabling T-cells. As a result, antagonists of these checkpoint proteins, known as immune checkpoint inhibitors, have recently begun to be recognized as the standard-of-care front-line treatment for increasing the efficacy of the body's immune system in fighting certain cancers [42]. In contrast to these cell and drug-based approaches, Dr. Mei Zhang has identified a product from nature, a set of molecules easily purified from oats, that also activates the immune system and eradicates cancer in animal models [43]. Currently, the therapy is being tested in dog trials of a bone disease common in animals called osteosarcoma [44]. This is considered a good model for the human disease, and if it is proven efficacious in this animal model, seeking approval to test the idea in osteosarcoma patients would be pursued [45]. The potential for these immune-type therapies to impact health and disease is in its infancy, and these congressionally mandated funds, although small, may have an outsized impact on health research due to their translational and interdisciplinary focus. To date, most immune and cell therapy treatments are focused on cancer, but emerging evidence suggests they may be effective in mediating a host of inflammatory conditions as well, further amplifying the potential impact of these developments [46]. Thus, the DOD support for cancer therapies may revolutionize many health care challenges facing veterans and society at large.

## CONCLUSION

Military medicine and medical research, supported by a combination of stakeholders including government, academia, hospitals, and industry, is a growing field that has significant overlap with society's healthcare needs. Increasingly, the needs of warfighters, support personnel, veterans, and society overlap with biomedical research as a nexus of interest and source of potential solutions. Whether fighting the next outbreak with a novel vaccine or finding potential treatments to cure cancer, collaborations around military medicine, such as those seen in Cleveland and across Ohio, are translating novel discoveries towards the dual needs of warfighters and society. Further, the themes of human performance and resilience provide frameworks for developing effective societal responses to challenges.
